# On the regulation, function, and localization of the DNA-dependent ATPase PICH

**DOI:** 10.1007/s00412-012-0370-0

**Published:** 2012-04-25

**Authors:** Manuel Kaulich, Fabien Cubizolles, Erich A. Nigg

**Affiliations:** 1 Growth & Development, Biozentrum, University of Basel, Klingelbergstrasse 50/70, 4065 Basel, Switzerland; 2 Present Address: Howard Hughes Medical Institute, Department of Cellular & Molecular Medicine, UCSD School of Medicine, 9500 Gilman Drive, La Jolla, CA 92093-0686 USA

## Abstract

The putative chromatin remodeling enzyme Plk1-interacting checkpoint helicase (PICH) was discovered as an interaction partner and substrate of the mitotic kinase Plk1. During mitosis PICH associates with centromeres and kinetochores and, most interestingly, constitutes a robust marker for ultrafine DNA bridges (UFBs) that connect separating chromatids in anaphase cells. The precise roles of PICH remain to be clarified. Here, we have used antibody microinjection and siRNA-rescue experiments to study PICH function and localization during M phase progression, with particular emphasis on the role of the predicted ATPase domain and the regulation of PICH localization by Plk1. We show that interference with PICH function results in chromatin bridge formation and micronucleation and that ATPase activity is critical for PICH function. Interestingly, an intact ATPase domain of PICH is required for prevention of chromatin bridge formation but not for UFB resolution, and quantitative analyses of UFB and chromatin bridge frequencies suggest that these structures are of different etiologies. We also show that the ATPase activity of PICH is required for temporal and spatial control of PICH localization to chromatin and that Plk1 likely controls PICH localization through phosphorylation of proteins distinct from PICH itself. This work strengthens the view that PICH is an important, Plk1-regulated enzyme, whose ATPase activity is essential for maintenance of genome integrity. Although not required for the spindle assembly checkpoint, PICH is clearly important for faithful chromosome segregation.

**Electronic supplementary material:**

The online version of this article (doi:10.1007/s00412-012-0370-0) contains supplementary material, which is available to authorized users.

## Introduction

The DNA-dependent SNF2/SWI ATPase helicase Plk1-interacting checkpoint helicase (PICH) was originally identified as a binding partner and substrate of polo-like kinase 1 (Plk1), a major regulator of M phase progression (Baumann et al. 2007). Whereas PICH is largely cytoplasmic during interphase, it concentrates in the centromere/kinetochore (KT) region of condensed chromosomes at the onset of mitosis. Most strikingly, PICH was discovered to decorate thin threads that frequently connect the KTs of sister chromatids during anaphase (Baumann et al. 2007; Wang et al. 2008). As many of these threads comprise centromeric DNA, it follows that disentanglement of sister chromatid centromeres through topoisomerase action is completed only after anaphase onset (Baumann et al. 2007; Spence et al. 2007; Wang et al. 2010, 2008). Although PICH-positive threads comprise DNA, they cannot readily be visualized by either DNA-intercalating dyes or anti-histone antibodies (Baumann et al. 2007). Thus, PICH has become the marker of choice for monitoring threads that are now commonly referred to as ultrafine DNA bridges (UFBs; Chan and Hickson 2011). Further interest in these structures has been triggered by the discovery that components of the BTR complex (composed of the Bloom syndrome helicase (BLM), TOP3A, and RMI1) co-localize with PICH on UFBs (Chan et al. 2007; Hutchins et al. 2010). Importantly, BTR complex association with UFBs requires binding to PICH (Ke et al. 2011), and not all PICH-positive UFBs carry the BTR complex (Chan et al. 2007). Furthermore, it has become clear that not all UFBs are derived from centromeres (Chan and Hickson 2011). In particular, a subpopulation of non-centromere-derived UFBs is characterized by co-localization of PICH with the Fanconi anemia proteins FANCD2 and FANCI (Chan et al. 2009a; Naim and Rosselli 2009). These Fanconi anemia proteins specifically associate with fragile site loci and presumably mark abnormally intertwined DNA structures induced by replication stress. The association of PICH with the BTR complex and Fanconi anemia proteins suggests that the processing and resolution of UFBs plays an important role in the maintenance of genome stability.

At a molecular level, the function of PICH is far from understood. An initial proposal that PICH might be required for the spindle assembly checkpoint (SAC) (Baumann et al. 2007) was subsequently challenged by the demonstration that the siRNA oligonucleotides used in this early study affected the SAC through an off-target effect on the essential SAC component Mad2 (Hubner et al. 2010). Other siRNA-based studies suggested that PICH is involved in the maintenance of chromosome architecture (Kurasawa and Yu-Lee 2010; Leng et al. 2008), but interpretation of these results is also complicated by possible off-target effects (Hubner et al. 2010). Most recently, purified recombinant PICH was shown to display nucleosome-remodeling activity (Ke et al. 2011), in line with properties expected for a member of the SNF2/SWI family of DNA-dependent ATPases. Data reported in the same study point to an attractive model according to which PICH and BLM cooperate to unravel chromatin and remove nucleosomes, in order to allow for resolution of catenated or aberrant DNA structures (Ke et al. 2011).

The functional significance of the interaction between PICH and the mitotic kinase Plk1 also remains to be fully understood. Inhibition or siRNA-mediated knockdown of Plk1 causes PICH to spread from centromeres/KTs over chromosome arms, suggesting that Plk1 normally functions to remove PICH from arms and allow its concentration at centromeres/KTs (Baumann et al. 2007; Leng et al. 2008; Santamaria et al. 2007). In return, PICH has been identified as a recruitment factor for Plk1 on chromosome arms (Leng et al. 2008; Santamaria et al. 2007). In the present study, we have combined antibody microinjection and siRNA-rescue experiments to study the function, regulation, and localization of PICH during M phase progression. In particular, we have explored the roles of the predicted ATPase domain, the possible etiological relationship between UFBs and chromatin bridges, and the mechanism through which Plk1 regulates PICH localization during M phase progression.

## Results

### PICH function is required for efficient chromosome segregation

**Fig. 1:**
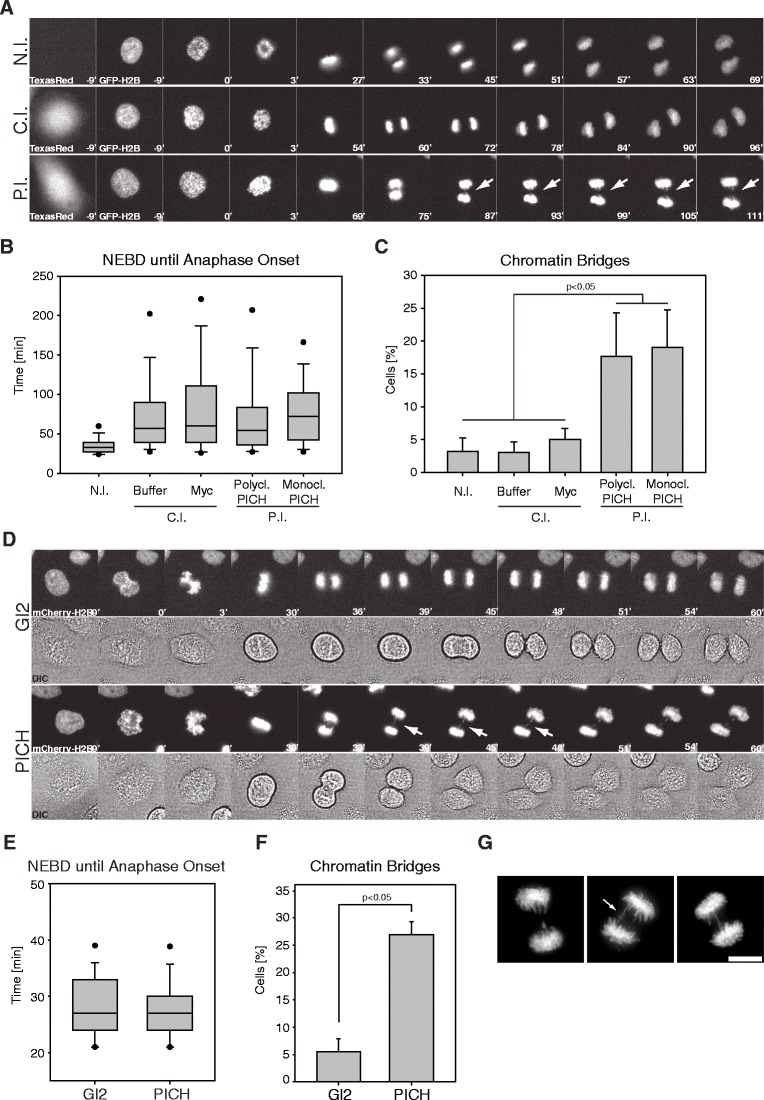
Anti-PICH antibody injection or siRNA-mediated PICH depletion causes anaphase chromatin bridges. **a** Representative stills of time-lapse videos showing HeLaS3 cells stably expressing histone 2B-GFP, after antibody microinjection (*N.I.* non-injected, *C.I.* control injected (buffer or Myc mAb), *P.I.* PICH injected (polyclonal or monoclonal antibodies)). TexasRed signal identifies microinjected cells. *T* = 0 was set at nuclear envelope breakdown (NEBD) and numbers indicate elapsed time (minutes). *Arrows* indicate chromatin bridge formation. **b** *Box-and-whisker plot* showing elapsed time (minutes) from NEBD to anaphase onset for individual microinjected cells. Analyses were performed on >120 cells per condition, over three independent experiments. *Lower and upper whiskers* represent 10th and 90th percentiles, respectively. **c** *Bar graph* showing the percentage of chromatin bridges after microinjection of the indicated antibodies. Analyses were performed on >120 cells per condition, over three independent experiments. Student’s *t* test revealed significance at *p* < 0.05. **d** Representative stills of time-lapse videos showing HeLaS3 cells stably expressing histone 2B-mCherry after transfection with the Gl2 (control) and PICH-directed siRNA oligonucleotides. *T* = 0 was set at NEBD and numbers indicate elapsed time (minutes). *Arrows* indicate chromatin bridge formation. **e** *Box-and-whisker plot* showing elapsed time (minutes) from NEBD to anaphase onset for individual siRNA-transfected cells. Analyses were performed on >120 cells per condition, over three independent experiments. *Lower and upper whiskers* represent 10th and 90th percentiles, respectively. **f** *Bar graph* showing the percentage of chromatin bridges after the indicated siRNA transfections. Analyses were performed on >120 cells per condition, over three independent experiments. Student’s *t* test revealed significance at *p* < 0.05. **g** Representative images of PTEMF fixed HeLaS3 anaphase cells, revealing typical chromatin bridges after PICH knockdown. Cells were stained for DNA with 4′-6-diamidino-2-phenylindole (*DAPI*). *Scale bar* represents 10 μm

Previous studies aimed at uncovering the function of PICH by siRNA had yielded inconsistent results (Baumann et al. 2007; Hubner et al. 2010; Kurasawa and Yu-Lee 2010; Leng et al. 2008). Thus, we resorted to antibody microinjection as a powerful, alternative approach for probing protein function. We raised and characterized a monoclonal antibody (mAb; clone 142-26-3) against human PICH, which recognized PICH in immunofluorescence microscopy, Western blotting, as well as immunoprecipitation (Fig. S1). Staining patterns matched those reported previously (Baumann et al. 2007; Wang et al. 2008), and antibody specificity was confirmed by siRNA-mediated knockdown of PICH (Fig. S1A, C). Furthermore, Plk1 was readily co-precipitated with PICH by mAb 142-26-3 (Fig. S1B), as expected (Baumann et al. 2007). To examine the consequences of introducing anti-PICH antibodies into living cells, HeLaS3 cells stably expressing histone 2B-GFP were synchronized in G1/S phase of the cell cycle (Sillje et al. 2006), before they were injected with mAb 142-26-3 or a previously characterized polyclonal anti-PICH Ab (Baumann et al. 2007). Control injections were performed using either injection buffer alone or a mAb specific for a Myc epitope (9E10; Evan et al. 1985). Using co-injected TexasRed for identification of injected cells, these were monitored by time-lapse video microscopy. None of the antibody injections detectably accelerated mitotic progression (Fig. 1a, b; videos 1–3), supporting the conclusion that PICH is not required for the establishment of a functional SAC (Hubner et al. 2010). To extend this conclusion, we also injected mitotically arrested HeLaS3 cells in which the SAC had been activated by a 5-h treatment with nocodazole. In this case, a function-neutralizing anti-Mad2 mAb served as a positive control (Fava et al. 2011). Cells were kept in culture for 3 h after injection, before they were fixed and analyzed by immunofluorescence microscopy. Whereas injection of the Myc mAb did not affect the SAC-induced mitotic arrest, injection of the anti-Mad2 mAb leads to the expected checkpoint override and premature mitotic exit (Fig. S2A, B). Importantly, neither mAb 142-26-3 nor the polyclonal anti-PICH Ab affected the nocodazole-induced SAC arrest (Fig. S2A, B), indicating that PICH is not required for maintenance of SAC activity.

The results of these microinjection experiments provide independent confirmation for our previous conclusion, inferred from siRNA, that PICH is not required for the SAC (Hubner et al. 2010). More interestingly, cells injected with anti-PICH Abs showed a clear phenotype with regard to the fidelity of chromosome segregation. In particular, we observed a striking increase in the frequency of chromatin bridges during anaphase (Fig. 1a, c; video 1–3). Whereas such bridges were seen in only 3 % of non-injected cells and 3–5 % of control injected cells (buffer or anti-Myc mAb, respectively), their frequency rose to nearly 20 % in response to anti-PICH antibody injection (mAb or polyclonal Ab, respectively) (Fig. 1c). In addition, anti-PICH antibody injection caused some increase in the frequency of individual chromosomes that failed to congress efficiently, but the duration of prometaphase was not significantly extended (data not shown). To corroborate these results through an antibody-independent approach, we also performed siRNA-mediated knockdown experiments in HeLaS3 cells stably expressing histone 2B-mCherry (Schmitz et al. 2010; Steigemann et al. 2009), followed by time-lapse video microscopy (Fig. 1d; videos 4–5). Using siRNA duplexes previously shown to be specific for PICH (Hubner et al. 2010) and thus not affecting the duration of M phase progression (Fig. 1e), we could demonstrate a prominent increase in the frequency of chromatin bridges in anaphase cells (27 %, as compared to 5 % in Gl2 transfected cells; Fig. 1f), and again, some chromosomes appeared to show delayed congression (data not shown). Analysis of fixed HeLaS3 cells showed that PICH depletion commonly resulted in the formation of one to three chromatin bridges per cell (Fig. 1g). In favorable images, chromatin bridges seemed to represent sister chromatids whose arms had apparently failed to separate (Fig. 1g; arrow in middle panel). Together, these results demonstrate that abrogation of PICH function by either antibody microinjection or siRNA-mediated knockdown does not affect the timing of mitotic progression but instead results in increased formation of chromatin bridges during anaphase. Importantly, the chromatin bridges of PICH-depleted cells could readily be visualized using fluorescently tagged histone 2B or DAPI, which clearly distinguishes them from the UFBs seen in PICH-positive cells (Baumann et al. 2007; Chan et al. 2007; Wang et al. 2010, 2008). Similar results on the phenotype of PICH-depleted cells were independently reported in two recent studies (Ke et al. 2011; Kurasawa and Yu-Lee 2010).

We also scored PICH-depleted cells for the formation of micronuclei (MN). Whereas only 0.9 % of control cells were found to harbor MNs, such structures could be seen in 4.2 % of PICH-depleted cells (Fig. S3A, B). This observation falls in line with recent results (Ke et al. 2011) and the notion that chromatin bridges often give rise to MN formation at the end of mitosis (Hoffelder et al. 2004; Naim and Rosselli 2009; Utani et al. 2010). As PICH depletion from proliferating cells causes formation of both chromatin bridges and micronuclei, we conclude that PICH is required for genome integrity.

### The ATPase activity of PICH is indispensable for genome stability

**Fig. 2:**
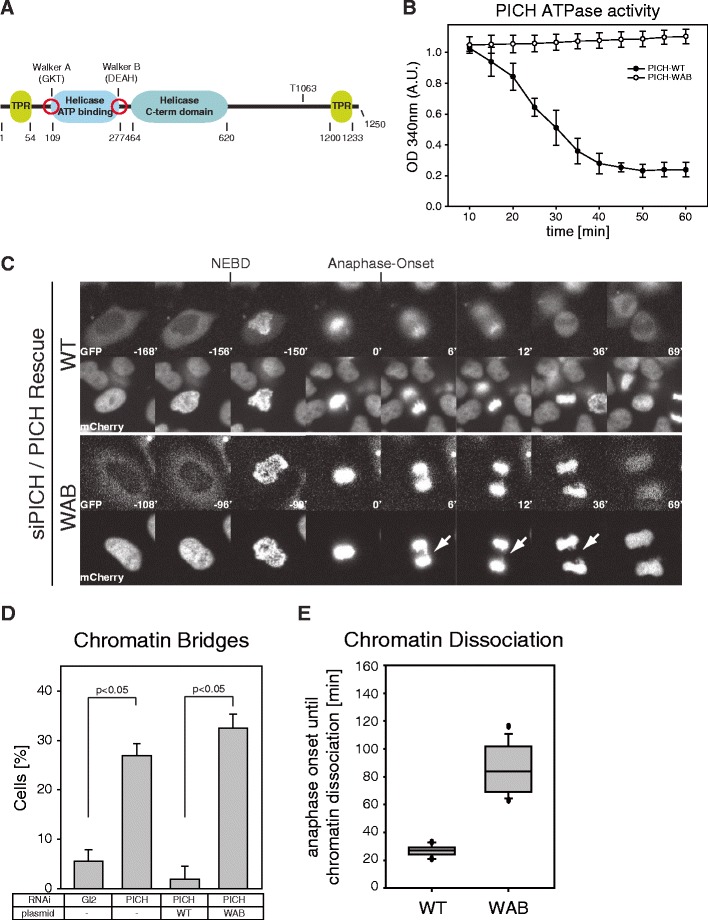
ATPase activity is critical for PICH function and localization. **a** Graphic illustrating the protein domains of PICH predicted by PROSITE (Sigrist et al. 2010), with numbers indicating the start and end of each domain (amino acid position). Walker A and B motifs (key residues in *brackets*) as well as the major Plk1 binding site (T1063) are *highlighted*. **b** *Line graph* showing the ATPase activity of PICH-WT and PICH-WAB. ATPase activity was measured as a decrease in OD 340 nm over time (minutes) and displayed in arbitrary units. **c** Representative stills of time-lapse videos showing HeLaS3 cells stably expressing histone 2B-mCherry after co-transfection with PICH siRNA duplexes and siRNA refractory PICH rescue plasmids. *T* = 0 was set at the onset of anaphase and numbers indicate elapsed time (minutes). Consistent with previous data (Hubner et al. 2010), the expression of PICH-WT and PICH-WAB increased the time required from NEBD until anaphase onset, due to sequestration of Plk1. *Arrows* indicate chromatin bridge formation. **d** *Bar graph* showing the percentage of anaphase cells showing chromatin bridges after treatment as in **c**. Analyses were performed on >30 cells per condition, over three independent experiments. Student’s *t* test revealed significance at *p* < 0.05. **e** *Box-and-whisker plot* showing elapsed time (minutes) from anaphase onset until dissociation from chromatin of PICH WT and WAB mutant, respectively, after treatment of cells as in **c**. Analyses were performed on >30 cells per condition, over three independent experiments. *Lower and upper whiskers* represent 10th and 90th percentiles, respectively

As predicted for a member of the SNF2/SWI class of ATPases, PICH carries Walker A and Walker B motifs that are implicated in phosphate binding and Mg^2+^ binding, respectively (Fig. 2a) (Baumann et al. 2007; Gorbalenya and Koonin 1993). To demonstrate the predicted ATPase activity of PICH and study its biological role, we generated a mutant (PICH-WAB) in which residues K128 (Walker A) and E229 (Walker B) were substituted by alanine (A) and glutamine (Q), respectively. Then, 293 T cells were transfected with PICH-WT or PICH-WAB and arrested in mitosis by addition of nocodazole, before the overexpressed PICH proteins were immunoprecipitated and analyzed for their ability to hydrolyze ATP in vitro. In contrast to PICH-WT, PICH-WAB did not show any in vitro ATPase activity (Fig. 2b), demonstrating that the ATPase domain of PICH is functional and requires the Walker A and B motifs. We recognize that the Walker A box substitution of lysine with alanine is likely to prevent nucleotide binding and not just hydrolysis and that many ATPases undergo a conformational change upon ATP binding (Walker et al. 1982). Thus, we emphasize that the effects reported in this study do not necessarily result from a failure of the WAB mutant to hydrolyze ATP but could also reflect an inability to undergo an ATP-dependent conformational change.

To test the importance of the ATPase activity of PICH for the preservation of chromosome integrity during mitosis, we carried out rescue experiments in siRNA-treated cells (Fig. 2c–e). HeLaS3 cells stably expressing histone 2B-mCherry were transfected with PICH-directed siRNA duplexes and siRNA-resistant (Fig. S4A) plasmids coding for PICH-WT and PICH-WAB, respectively, before traverse of mitosis was analyzed by time-lapse video microscopy (Fig. 2c, d; videos 6–7). The expression of PICH-WT readily suppressed the formation of chromatin bridges during mitosis (to 2 %), whereas PICH-WAB failed to restore fidelity in chromosome segregation (with 32 % of cells showing bridges). This indicates that the ATPase activity of PICH is critical for preventing the formation of chromatin bridges during anaphase and hence for PICH function in vivo.

### ATPase activity contributes to the regulation of PICH localization

**Fig. 3:**
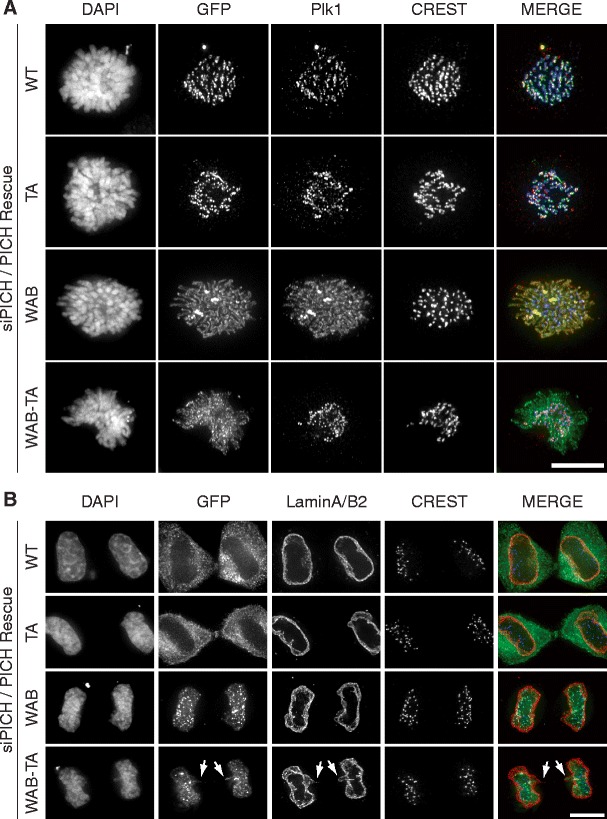
Effect of ATPase activity on PICH localization. HeLaS3 cells were transfected with PICH siRNA and plasmids coding for GFP-tagged PICH WT and mutant proteins, before they were fixed with PTEMF and analyzed by immunofluorescence, as indicated. WAB mutants lack ATPase activity, while TA mutants lack the major Plk1 docking site (T1063). DAPI was used to stain DNA. *Scale bar* represents 10 μm. Representative images show prometaphase cells (**a**) and late telophase cells (**b**)

In the course of the above experiments, we noticed that the ATPase activity of PICH is critical for PICH localization at the end of mitosis (Fig. 2c, e). Whereas PICH-WT dissociated from chromatin during telophase and prior to chromatin decondensation (on average 26 min after anaphase onset), consistent with earlier results (Baumann et al. 2007; Leng et al. 2008), PICH-WAB persisted on chromatin even after the onset of chromatin decondensation (for ca. 90 min after anaphase onset) (Fig. 2e; videos 6–7). This prompted us to examine the influence of the ATPase motif on the subcellular localization of PICH more carefully. To this end, GFP-tagged versions of PICH were expressed in HeLaS3 cells that had been depleted of endogenous PICH by siRNA. During early mitosis, GFP-tagged PICH-WT showed the expected concentration at centromeres/kinetochores (Fig. 3a). In stark contrast, PICH-WAB was found to spread all over the chromosome arms (Fig. 3a). In line with the results shown above (Fig. 2c, e), major differences in localization were also seen at the end of mitosis (Fig. 3b). GFP-PICH-WT was excluded from the re-forming nuclei during telophase and then persisted in the cytoplasm until the next G2/M transition, just like the endogenous protein. In contrast, the GFP-tagged PICH-WAB protein remained associated with chromatin and KTs until after reformation of nuclear envelopes (Fig. 3b), before it was extruded from the nucleus at the end of mitosis (video 7). Together, these results demonstrate that the ATPase activity of PICH influences the chromatin association of this protein during both early and late stages of mitosis.

The same dependency of PICH localization on the Walker A/B motifs was also observed when analyzing mutants (PICH-TA, with T1063 changed to A) that are unable to recruit the mitotic kinase Plk1 (Fig. S4B; Baumann et al. 2007). Compared to the ATPase active TA version of PICH (PICH-TA), the WAB version (PICH-WAB-TA) spread to chromosome arms during early mitosis (Fig. 3a), and it also showed delayed dissociation from chromatin during late mitosis (Fig. 3b) (videos 8–9). Plk1 closely followed the re-localization of the binding-competent PICH-WAB from kinetochores to chromosome arms, but no such re-localization was observed in the case of the binding-incompetent PICH-WAB-TA (Fig. 3a). These results demonstrate that the ATPase activity is critical for determining PICH localization, and they confirm that PICH is a major interaction partner of Plk1 on mitotic chromosomes (Baumann et al. 2007; Hutchins et al. 2010; Leng et al. 2008; Santamaria et al. 2007). Interestingly, expression of PICH-TA in the PICH siRNA background reduced the frequency of chromatin bridges to a similar extent as expression of PICH WT (video 8 and data not shown), indicating that Plk1 binding to PICH is not required to suppress chromatin bridge formation. In contrast, expression of PICH-WAB-TA was unable to suppress the PICH-depletion-induced chromatin bridge formation, again illustrating the need for ATPase activity to rescue this phenotype (Fig. 3b, arrows; video 9).

### On the relationship between UFBs and chromatin bridges

**Fig. 4:**
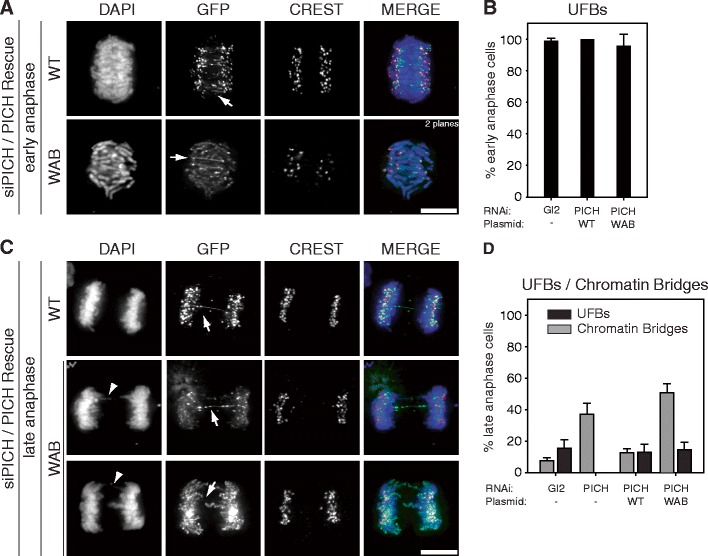
Chromatin bridges and UFBs are likely of distinct origin. HeLaS3 cells were transfected with PICH siRNA and plasmids coding for GFP-tagged PICH WT and PICH-WAB, before they were fixed with PTEMF and analyzed by immunofluorescence, as indicated. Representative images show early (**a**) and late anaphase (**c**) cells with UFBs and chromatin bridges marked by *arrows* and *arrowheads*, respectively. Anaphase cells were monitored for the presence of UFBs and/or chromatin bridges, and the frequencies of these structures are shown in histograms **b** and **d** for early or late anaphase cells, respectively. Histograms represent the averages of three independent experiments. Analyses were performed on >100 cells per condition, over three independent experiments. Student’s *t* test revealed significance at *p* < 0.05. *Scale bar* represents 10 μm

The above siRNA-rescue experiments also provided an opportunity to explore the quantitative relationship between UFBs and chromatin bridges. Whereas UFBs are commonly observed in undisturbed cells (Baumann et al. 2007; Wang et al. 2008), chromatin bridges are prominent only upon PICH depletion (Ke et al. 2011; Kurasawa and Yu-Lee 2010; Leng et al. 2008; this study). To clarify the relationship between the two structures, we counted the number of anaphase cells showing UFBs and/or chromatin bridges after replacement of endogenous PICH by either PICH-WT or PICH-WAB (Fig. 4). UFBs could readily be detected in virtually all early anaphase cells, regardless of whether PICH-WT or PICH-WAB was used to rescue PICH depletion (Fig. 4a, arrows and b). In the majority of late anaphase cells, UFBs had been resolved, as expected (Baumann et al. 2007; Wang et al. 2008). However, the proportion of residual cells still showing UFBs was remarkably similar (10–15 %; Fig. 4c), regardless of whether PICH-WT or PICH-WAB was expressed in the rescue experiments (Fig. 4d). In striking contrast, the number of late anaphase cells showing chromatin bridges was much higher when rescue was attempted by PICH-WAB than PICH-WT (51 % compared to 14 %; Fig. 4c, d). These results argue that the ATPase activity of PICH is critical to prevent the formation of chromatin bridges but not for the resolution of UFBs.

### How does Plk1 regulate PICH localization?

**Fig. 5:**
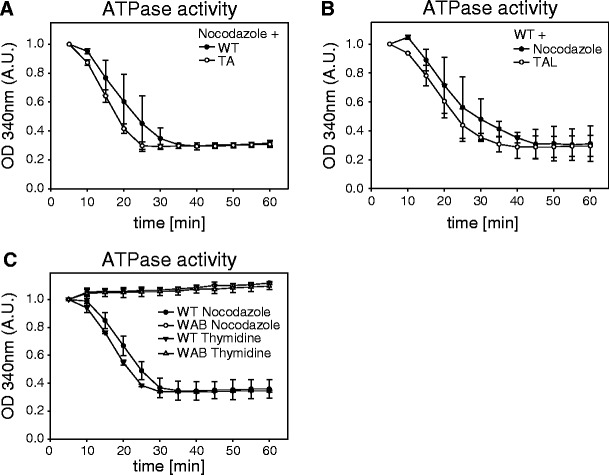
Plk1 does not regulate PICH ATPase activity. PICH proteins were isolated from cells and ATPase activity measured in vitro. All *line graphs* show ATPase activity, measured as decrease in OD 340 nm over time (minutes) and displayed in arbitrary units. **a** ATPase activity of PICH-WT and PICH-TA isolated from nocodazole-arrested 293 T cells. **b** ATPase activity of PICH-WT isolated from prometaphase cells with active Plk1 (nocodazole arrest) or inactive Plk1 (TAL-induced arrest). **c** ATPase activity of PICH-WT and PICH-WAB isolated from nocodazole or thymidine-arrested cells

In previous studies, we had shown that depletion or inhibition of Plk1 during early mitosis results in retention of PICH on chromosome arms (Baumann et al. 2007; Santamaria et al. 2007). Our present data show that mutations in the ATPase domain also cause a spreading of PICH from centromeres/kinetochores to chromosome arms (Fig. 3a). This prompted the question of whether Plk1 might regulate PICH localization via inhibition of its ATPase activity. Arguing against this possibility, we found no significant difference in the ATPase activity of PICH-WT and PICH-TA (Fig. 5a), although the latter protein is unable to recruit Plk1 and not phosphorylated by Plk1 to any major extent (Fig. S4B; Baumann et al. 2007). Furthermore, addition of the Plk1 inhibitor TAL did not detectably affect the ATPase activity of PICH-WT (Fig. 5b). In line with the conclusion that Plk1 does not regulate the ATPase activity of PICH, near-constant ATPase activities were measured after isolating PICH from cells at different cell cycle stages (Fig. 5c). In these experiments, tagged PICH-WT (or PICH-WAB for control) was isolated from 293 T cells that had been synchronized in mitosis or G1/S by treatment with nocodazole or thymidine, respectively, and cell cycle stages were confirmed by analysis of phosphohistone H3 staining (a marker for mitosis; Crosio et al. 2002) and responsiveness of PICH to Plk1 binding (Fig. S4C). In parallel, phosphorylation of WT and mutant PICH was confirmed by co-transfections with a constitutively active Plk1 mutant (Plk1-T210D; Jang et al. 2002) and monitoring of a typical electrophoretic upshift in PICH (Baumann et al. 2007) (Fig. S4C).

**Fig. 6:**
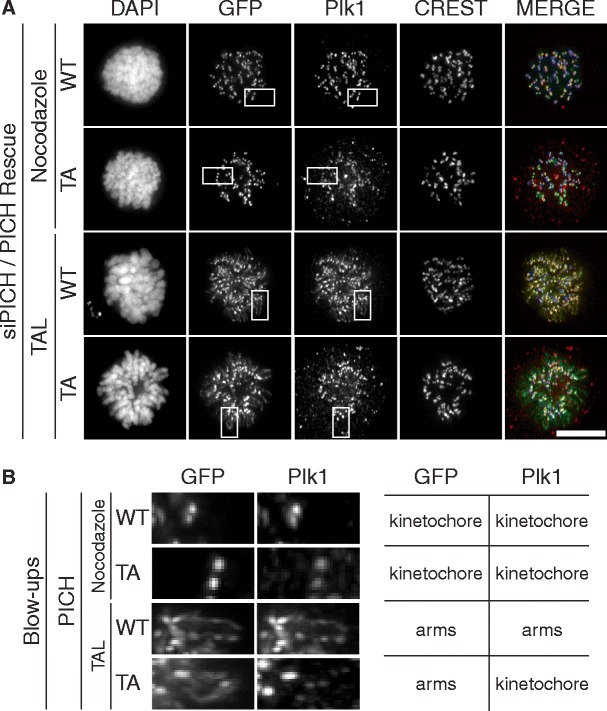
Plk1-mediated removal of PICH from chromosome arms is independent of Plk1 binding. HeLaS3 cells were transfected with plasmids coding for GFP-tagged PICH WT or TA mutant (defective in Plk1 binding) proteins, before they were fixed with PTEMF and analyzed by immunofluorescence, as indicated. Cells were arrested in prometaphase by addition of nocodazole or the Plk1-inhibitor TAL. **a** Representative images of prometaphase HeLaS3 cells showing PICH and Plk1 localization, depending on the activity state of Plk1 and the Plk1-binding ability of PICH. *Scale bar* represents 10 μm. **b** Blow-ups of the *squared boxes* shown in **a**. Note that both PICH WT and PICH-TA spread over chromosome arms in response to Plk1 inhibition; Plk1 co-distributes over chromosome arms with PICH WT but not PICH-TA

In a final series of experiments, we asked whether Plk1 controls PICH localization through phosphorylation of PICH itself or some other protein. To this end, HeLaS3 cells were co-transfected with siRNA oligonucleotides targeting endogenous PICH (Hubner et al. 2010) and siRNA-resistant plasmids coding for PICH-WT or PICH-TA, respectively. Following pre-synchronization with thymidine, cells were released and arrested in mitosis by the addition of either nocodazole or the Plk1-inhibitor TAL (Santamaria et al. 2007), both of which cause a spindle checkpoint-dependent prometaphase arrest. The localizations of PICH-WT and PICH-TA as well as Plk1 were then examined, with particular emphasis on the dependencies of these localizations on Plk1 inhibition (Fig. 6a, b). In nocodazole-arrested cells, all three proteins co-localized at the centromere/kinetochore region of mitotic chromosomes, as expected (Baumann et al. 2007; Leng et al. 2008; Santamaria et al. 2007). In response to Plk1 inhibition, PICH-WT redistributed to chromosome arms, consistent with the notions that PICH is a major binding partner of Plk1 on chromosome arms and that Plk1 normally removes PICH from arms (Leng et al. 2008; Santamaria et al. 2007). Remarkably, the PICH-TA mutant showed the same re-localization to chromosome arms in response to Plk1 inhibition (Fig. 6a, b), in spite of the fact that this mutant cannot bind Plk1 and thus represents a poor substrate of this kinase (see also Fig. S4B). This result would not have been expected if PICH removal from chromosome arms was triggered by Plk1 recruitment to PICH and subsequent PICH phosphorylation. Thus, it seems likely that phosphorylation of an as-yet unidentified substrate by Plk1 is required to remove PICH from chromosome arms and cause its concentration at the centromere/kinetochore region.

## Discussion

Using a combination of antibody microinjection and siRNA-rescue experiments, we have explored the function of PICH during M phase progression as well as the mechanisms that contribute to control its localization at centromeres/KTs and chromosome arms, respectively. Neither inactivation of PICH through microinjection of monoclonal or polyclonal antibodies nor PICH depletion through siRNA significantly affected the timing between nuclear envelope breakdown and anaphase onset. This demonstrates that PICH is not required for either establishment or maintenance of the SAC, confirming and extending our earlier conclusion (Hubner et al. 2010). However, inactivation or depletion of PICH clearly reduced the fidelity of chromosome segregation. In response to both antibody microinjection and siRNA-mediated PICH depletion, we observed a substantial increase in the frequency of chromatin bridges and micronuclei formation. Similar results were recently reported in an independent study on the phenotypic consequences of siRNA-mediated PICH depletion (Ke et al. 2011). In this latter study, it was proposed that the chromatin bridges observed in PICH- or BLM-depleted cells essentially reflect the reacquisition of histones by PICH-positive UFBs (Ke et al. 2011). A priori, this is an attractive interpretation, in line with the author’s model that PICH and BLM cooperate to remove nucleosomes from anaphase threads. However, we emphasize that in the present study, chromatin bridges were observed in only 25–50 % of cells from which PICH had been depleted (or in which it had been functionally inactivated by antibody injection), while, in contrast, PICH-positive UFBs are seen in nearly a 100 % of dividing PICH-positive cells (Baumann et al. 2007). Furthermore, our siRNA and rescue experiments suggest that the ATPase activity of PICH is much more critical to prevent the formation of chromatin bridges than the resolution of UFBs. The near-constant frequency of UFBs under different experimental conditions and the strikingly distinct responses of UFBs and chromatin bridges to PICH-WAB are difficult to reconcile with the recent proposal that impaired PICH functionality results in a conversion of UFBs to chromatin bridges (Ke et al. 2011).

At first glance, it seems surprising that the ATPase activity of PICH is apparently not required for efficient resolution of UFBs. One obvious caveat to this conclusion is that sufficient levels of endogenous PICH may resist siRNA-mediated depletion to help catalyze UFB resolution (but not to prevent chromatin bridge formation). Alternatively, UBF resolution may be more dependent on PICH binding to UFBs than ATPase activity. According to this scenario, UBF resolution would likely be catalyzed by proteins that depend on PICH for their recruitment to UFBs.

The mechanism through which PICH activity contributes to the maintenance of chromosome segregation fidelity remains to be fully understood. According to one interesting recent proposal, PICH cooperates with the BLM helicase to unravel chromatin, thereby creating nucleosome-free areas, so that catenated or aberrant DNA structures can be resolved (Ke et al. 2011). In other studies, the possibility has been emphasized that PICH may act as a chromatin remodeling enzyme to facilitate the action of topoisomerase II (Wang et al. 2010, 2008). Whatever the precise role of PICH, our current results clearly indicate that this function requires its ATPase activity. In fact, wild-type PICH readily rescued the PICH-depletion phenotype, but a WAB mutant version (lacking ATPase activity as a consequence of mutations in the Walker A and Walker B motifs) failed to do so.

We also discovered that the ATPase activity of PICH contributes to regulate the localization of PICH, particularly its association with chromatin. In an earlier study, we had already shown that a PICH mutant carrying a substitution of the tripeptide GKT within the Walker A motif by triple alanine failed to localize to chromatin (Baumann et al. 2007). In light of the results reported here, we conclude that this GKT to AAA mutation presumably caused a seriously deleterious effect on overall protein structure. In fact, we now found that a more subtle PICH mutant (PICH-WAB) in which residues K128 (Walker A) and E229 (Walker B) were substituted by alanine (A) and glutamine (Q), respectively, is still able to associate with chromatin. This falls in line with results reported earlier for a K128 PICH mutant (Leng et al. 2008). Interestingly, however, the mutations in PICH-WAB markedly affected the extent and timing of chromatin association. In contrast to PICH WT, which was concentrated on centromeres/KTs during early mitosis, the impairment of ATPase catalytic activity caused PICH-WAB to spread all over chromosome arms. Likewise, at the end of mitosis, PICH WT dissociated from chromatin concomitant with reformation of the nuclear envelope, but PICH-WAB showed a striking delay in its dissociation from decondensing chromatin. Regardless of whether the observed phenotypes reflect the inability of PICH-WAB to bind and hydrolyze ATP or undergo a conformational change in response to ATP binding, they strongly suggest that ATPase activity contributes to the release of PICH from chromatin.

The association of the PICH-WAB mutant with chromosome arms in prometaphase cells was highly reminiscent of the localization of PICH WT in response to Plk1 inhibition or depletion. However, our data provide no evidence to suggest that Plk1 regulates PICH through modulation of its ATPase activity. No differences could be observed when comparing the ATPase activity associated with PICH WT and the PICH-TA mutant that is unable to recruit Plk1, and no effect on PICH ATPase activity was seen after isolation of the protein from cells that had been treated with the Plk1 inhibitor TAL. Finally, a comparison between PICH isolated from S- or M-phase arrested cells revealed no differences in ATPase activity, arguing that PICH activity is either not regulated through the cell cycle or that regulation occurs through a mechanism that does not resist immunoprecipitation.

A rise in Plk1 activity during early mitosis causes the release of PICH from chromosome arms, resulting in the concentration of PICH at centromeres/kinetochores (Baumann et al. 2007; Santamaria et al. 2007). Plk1 recruitment to PICH is triggered by phosphorylation of a key residue (T1063) within PICH by cyclin-dependent kinase 1, which then results in extensive PICH phosphorylation by Plk1 (Baumann et al. 2007). In light of these early results, it seemed plausible that recruitment of Plk1 and consequent phosphorylation of PICH by Plk1 is the mechanism through which Plk1 triggers the dissociation of PICH from chromosome arms. However, this simple mechanism fails to explain why PICH-TA localizes predominantly to centromeres/kinetochores in early M phase (this study and Leng et al. 2008), unless it is overexpressed (Baumann et al. 2007), and it also fails to explain our present observation that the response of PICH-TA to Plk1 inhibition is indistinguishable from that of PICH WT. Upon Plk1 inhibition, both PICH WT and PICH-TA spread over chromosome arms, although the latter protein is unable to recruit Plk1 and serve as an efficient substrate for Plk1. Since these experiments were conducted in a PICH siRNA background, the results cannot be explained by dimerization between PICH-TA and endogenous PICH. We have considered the possibility that dissociation of PICH from chromosome arms might depend on Plk1-mediated removal of arm cohesin but the results obtained so far do not support this view (unpublished data). We thus find it difficult to avoid the conclusion that Plk1 regulates PICH localization through phosphorylation of as-yet unidentified substrates.

Complementing other recent studies (Chan and Hickson 2011; Hubner et al. 2010; Ke et al. 2011; Wang et al. 2010), the data reported here contribute to clarify several important aspects of PICH function, regulation, and localization. First we confirm that interference with PICH does not affect the functionality of the SAC. Nevertheless, PICH is clearly important for the fidelity of chromosome transmission. In the absence of PICH, the frequency of chromatin bridges as well as micronuclei increases substantially, indicating that PICH is important for maintenance of genome stability. We also show that PICH is an active ATPase, as predicted from its structure, and that ATPase activity is required for the mitotic function of PICH. Moreover, ATPase activity has profound spatial and temporal effects on the chromatin association of PICH. Finally, we could readily confirm that Plk1 regulates the association of PICH with chromatin. Surprisingly, however, our data indicate that dislocation of PICH from chromosome arms requires phosphorylation of proteins other than PICH itself. In the future, it will be interesting to identify the Plk1 substrate(s) that control(s) the temporal and spatial association of PICH with chromatin. Another challenge will be to understand the precise molecular function of PICH in chromatin remodeling and chromosome segregation.

## Materials and methods

### Antibodies

The monoclonal antibody (mAb; clone 124-26-3, IgG1 kappa) against PICH was raised against a truncated version (aa79-aa752) of the human recombinant His-PICH protein expressed in *Escherichia coli* (NCBI reference sequence: NP_060139.2). Other antibodies used in this study were described previously, i.e., rabbit anti-PICH (Baumann et al. 2007), mouse mAb anti-Mad2 and mAb anti-Myc (Chan et al. 2009b), mouse Plk1 mAb (Hanisch et al. 2006), or purchased from the following sources: mouse anti-alpha-tubulin (Sigma-Aldrich, St. Louise, MO, USA), human CREST autoimmune serum (Immunovision, Springdale, AR, USA), mouse anti-cyclinB1 (Millipore, Billerica, MA, USA), mouse anti-GFP mAb (kindly provided by Andreas Uldschmidt), and rabbit IgG (ab37415-5, Abcam, Cambridge, MA, USA). Prior to use, all mAbs were purified using HiTrap Protein G HP columns (GE Healthcare, Waukesha, WI, USA). For immunofluorescence analysis, primary antibodies were detected using Cy2-, Cy3-, or Cy5-conjugated donkey anti-mouse, anti-rabbit, or anti-human IgGs (Dianova, Hamburg, Germany). The secondary antibody used for Western blot analysis was goat anti-mouse (Bio-Rad, Hercules, CA, USA).

### Antibody microinjection

Antibody microinjection was performed using the FemtoJet system equipped with Femtotips II purchased from Eppendorf (Hamburg, Germany) connected to an Axiovert S100 microscope from Zeiss (Jena, Germany). Antibody concentration was set to 1 mg/ml in PBS, and 1.66 mg/ml TexasRed Dextran (Invitrogen, Grand Island, NY, USA) was added to all samples. Positive injection was achieved with injection times varying between 0.2 and 0.4 s, capillary pressure of 30–40 hPa, and injection pressure between 15 and 20 hPa. Prior to injection, cells were seeded either onto 35-mm μ-Dishes with grids from Ibidi (Martinsried, Germany) for time-lapse video microscopy or onto coverslips for PTEMF fixation and indirect immunofluorescence microscopy.

### Cloning procedures

For cloning of His-hPICH (aa79-aa752), a plasmid coding for human PICH was used (Baumann et al. 2007) together with DNA oligonucleotides 5′-ACAGATGTGTGCAACTCTGGCTTG-3′ and 5′-AGGCTGAGGCTGTGGTTTATTCAA-3′ for in-frame cloning into a pET-28a(+) vector (Novagen, Madison, WI, USA). N-terminally tagged GFP versions of PICH-WT and PICH-TA were previously described (Baumann et al. 2007). To generate PICH-WAB and PICH-WAB-TA, the following DNA oligonucleotides were used: K128A: 5′-gatgatatgggattaggggcgactgttcaaatcattgct-3′, E229Q: 5′-tatgtcatcctcgatcaagcacataaaataaaa-3′, and T1063A: 5′-CAATTTGATGCTTCAGCTCCCAAAAATGACATC-3′ with the QuickChange Site-Directed Mutagenesis kit (Stratagene, Santa Clara, CA, USA). To generate plasmids refractory to PICH siRNA duplexes, a series of seven silent point mutations was introduced using the DNA oligonucleotide 5′-GTTTTTCTGCTTACCACTCAGGTGGGAGGAGTGGGATTGACATTAACTGCAGCA-3′ and its corresponding antisense. Underlined letters correspond to the introduced point mutations.

### Cell culture and synchronization

HeLaS3 cells were cultured at 37 °C, in a 5 % CO2 atmosphere in DME media (Invitrogen, Grand Island, NY, USA), supplemented with 10 % heat-inactivated fetal calf serum and penicillin/streptomycin (100 and 100 μg/ml, respectively). Nocodazole (100 ng/ml), monastrol (150 μM), puromycin (0.5 μg/ml), and thymidine (2 mM) were obtained from Sigma-Aldrich (St. Louise, MO, USA). MG132 (10 μM) was obtained from EMD (Merck KGaA, Darmstadt, Germany). Prior to antibody injection, cells were synchronized by 24 h incubation in 2 mM thymidine, followed by release for 10 h (microscopic inspection confirmed that these cells were in G2 rather than M phase).

### Cell extracts, immunoprecipitation, and western blot analysis

Preparation of lysates and western blot analyses were performed as described previously (Sillje et al. 2006) using Tris lysis buffer (50 mM Tris–HCl pH 7.5, 150 mM NaCl, 1 % IGEPAL CA-630) containing 20 mM NaF, 20 mM β-glycerophosphate, 0.3 mM Na-vanadate, 20 μg/ml RNase A, 20 μg/ml DNase supplemented fresh with 1 mM Pefabloc SC (Roth), and 1/100 phosphatase inhibitor cocktail 1 + 2 (Sigma-Aldrich, St. Louise, MO, USA). Immunoprecipitation of overexpressed PICH was carried out using 0.5 μg of corresponding antibody and 20 μL Affi-Prep protein A agarose beads (Bio-Rad, Hercules, CA, USA) for 2 h at 4 °C, followed by extensive washing with lysis buffer and PBS. Where indicated, immunoprecipitates were subjected to western blot analyses.

### Enzyme-coupled ATPase assay

To monitor the ATPase activity of PICH, the following reaction solution was prepared: 25 mM triethanolamine pH 7.5, 13 mM Mg-acetate, 1.8 mM dithiothreitol, 5 mM ATP, 100 μg/ml bovine serum albumin, 3 mM phosphoenolpyruvate, 20 U/ml pyruvate kinase, and 20 U/ml lactic dehydrogenase (Sigma-Aldrich, St. Louise, MO, USA) (modified from Huang and Hackney 1994). To assay the ATPase activity of PICH, the protein was provided as an immunoprecipitate, linked to protein A agarose (Bio-Rad, Hercules, CA, USA), and analyzed in a total reaction volume of 125 μl, using a Ultrospec 3100 pro (GE Healthcare, Waukesha, WI, USA). ATPase activity was measured by monitoring the decrease of OD 340 nm, indicating conversion of NADH to NAD^+^. To this end, an initial OD 340 nm was set to 2.5–3.0 by addition of NADH (20 mg/ml), and samples were analyzed every 5 min for a total reaction time of 1 h. To obtain optimal results, the reaction mixture was gently stirred by pipetting up and down every 5 min. Graphical and statistical analyses were performed using SigmaPlot 11 (Systat Software Inc., Chicago, IL, USA).

### Immunofluorescence microscopy

Cells were seeded and grown on coverslips, treated as indicated, and simultaneous fixed and permeabilized for 10 min at RT with PTEMF buffer, as described previously (Chan et al. 2009b). Primary and secondary antibodies used in this study are listed above. DNA was stained with DAPI (2 μg/ml). Images were acquired using a DeltaVision Olympus IX71 microscope equipped with a ×60/1.42 oil objective. Collection and processing of acquired images was carried out using Softworx (Applied Precision, Issaquah, WA, USA).

### Transient plasmid and siRNA transfection

Plasmid transfection was performed using TransIT-LT1 reagent (Mirus Bio Corporation LLC, Madison, WI, USA) according to the manufacturer’s instructions. siRNA duplexes were transfected using Oligofectamine (Invitrogen, Grand Island, NY, USA) as described previously (Elbashir et al. 2001). The following siRNA duplexes were used and purchased from QIAGEN: Gl2 (Elbashir et al. 2001) and PICH-3 (Hubner et al. 2010). All plasmid and siRNA transfections were performed for 48 to 72 h with cells synchronized for 24 h in the presence of 2 mM thymidine.

### Time-lapse video microscopy

HeLaS3 cells stably expressing histone 2B-GFP (Sillje et al. 2006) or histone 2B-mCherry (Steigemann et al. 2009) were seeded in eight-well chambers from Ibidi (Martinsried, Germany), treated as indicated and imaged using a Nikon ECLIPSE Ti microscope equipped with a CoolLED pE-1 excitation system and a ×20/0.75 air Plan Apo objective (Nikon, Tokyo, Japan). Images were acquired at multiple positions every 3 min. Following antibody microinjections, GFP fluorescence and DIC images were acquired at each time point with 20- and 2-ms exposure times, respectively. TexasRed fluorescence was imaged every fifth time point with a 40-ms exposure. For siRNA knockdown and rescue experiments, histone 2B-mCherry fluorescence was acquired at every time point with a 20-ms exposure time. DIC images were collected as described above. To collect and process data, MetaMorph 7.7 software (MDS Analytical Technologies, Sunnyvale, CA, USA) in combination with SigmaPlot 11 (Systat Software Inc., Chicago, IL, USA) was used.

## Electronic supplementary material

Below is the link to the electronic supplementary material.

Figure S1 Characterization of monoclonal anti-PICH antibody (mAb). **A** Western blot analysis of lysates prepared after transfection of HeLaS3 cells with control (Gl2) or PICH-directed siRNA. The strong reduction of the PICH band (175 kDa) in response to PICH siRNA documents specificity of mAB 124-26-3, while blotting for alpha-tubulin confirms equal protein loading. **B** Immunoprecipitation (IP)–Western blot experiment showing that mAb 124-26-3 is able to immunoprecipitate endogenous PICH by from a lysate of nocodazole-arrested HeLaS3 cells; IP by an anti-GFP antibody was carried out as negative control. Immunoprecipitates were then subjected to western blotting using mAb 124-26-3 and Plk1, which is known to co-immunoprecipitate with PICH (Baumann et al. 2007). **C** Representative images of HeLaS3 prometaphase cells stained with the indicated antibodies. Prior to staining, cells were subjected to siRNA treatment, using the siRNA duplexes indicated to the *left*. Note that PICH-directed siRNA abolishes staining by the anti-PICH mAb, while depletion of Sgo1 causes this mAb to stain characteristic PICH-positive ultrafine DNA bridges, consistent with previous data (Baumann et al. 2007). *Scale bar* represents 10 μm (AI 2053 kb)

Figure S2 PICH mAb injection does not abolish a functional SAC. **A** HeLaS3 cells were grown asynchronously and supplemented with nocodazole for 5 h. Then, mitotic cells were injected with the indicated antibodies and kept 3 h in culture before PTEMF fixation. Cells were stained for DNA (DAPI) and TexasRed signal (*red*) was used to identify injected cells. *Scale bar* represents 10 μm. **B** Bar graph shows the percentages of injected cells that remained arrested in mitosis. Note that only anti-Mad2 injection abolished the SAC (positive control). Analyses were performed on >120 cells per condition, over three independent experiments. Student’s *t* test revealed significance at *p* < 0.05 (AI 2075 kb)

Figure S3 PICH depletion results in micronuclei formation. **A** HeLaS3 cells were transfected for 48 h with either Gl2 or PICH-directed siRNA duplexes. Then, they were subjected to PTEMF fixation and stained for DNA (DAPI). *Arrowheads* point to micronuclei that arose after PICH depletion. *Scale bar* represents 10 μm. **B** *Bar graph* demonstrates the percentages of micronuclei. Analyses were performed on >100 cells per condition, over three independent experiments. Student’s *t* test revealed significance at *p* < 0.05 (AI 1236 kb)

Figure S4 Biochemical properties of PICH mutants. **A** Evidence for siRNA resistance of PICH plasmids carrying siRNA refractory mutations. HeLaS3 cells were co-transfected with the indicated siRNA duplexes and PICH encoding plasmids, before they were probed by western blotting for expression of PICH and alpha-tubulin (as loading control). Note that introduction of seven silent point mutations (see “Materials and methods”) made PICH resistant to PICH-directed siRNA. *Numbers* indicate molecular weights (in kilodaltons). **B** Western blot analysis demonstrating the ability of different PICH mutants to interact with endogenous Plk1. Two hundred ninety-three T cells were transfected for 36 h with the indicated plasmids and synchronized in mitosis with nocodazole. Radioimmunoprecipitation assay lysates were then subjected to IP by anti-GFP antibodies and lysates as well as immunoprecipitates were probed by western blotting using the antibodies indicated. Note that binding of PICH to Plk1 is abolished upon mutation of threonine 1063 to alanine (*TA*). alpha-Tubulin was probed to show equal input, and numbers indicate molecular weights (in kilodaltons). **C** Western blot analysis was used to monitor the gel electrophoretic mobility of different PICH mutant proteins. Two hundred ninety-three T cells expressing the indicated proteins (PICH WT, PICH-TA, or PICH-WAB as well as constitutively active Plk1; Plk1-T210D-Flag) were synchronized by addition of thymidine, nocodazole, or TAL, a small molecule inhibitor of Plk1 (Santamaria et al. 2007). Phosphohistone 3 was used to monitor synchronization of cells in mitosis, and Flag and alpha-tubulin were probed to reveal equal expression of Plk1-T210D and equal loading, respectively. *Arrows* point to the slower migrating, phosphorylated species of PICH (p-PICH), and the faster migrating, un-phosphorylated form (PICH). *Numbers* indicate molecular weights (in kilodaltons) (AI 1370 kb)

Video 1 *N.I.* non-injected cell. Time-lapse video microscopy of a representative HeLaS3 cell, stably expressing histone 2B-GFP. Images were acquired at multiple positions every 3 min. GFP fluorescence and DIC images were acquired at each time point with 20- and 2-ms exposure times, respectively (AVI 480 kb)

Video 2 *C.I.* control injected cell. Time-lapse video microscopy showing a representative HeLaS3 cell, stably expressing histone 2B-GFP, injected with either buffer or Myc mAb. Images were acquired at multiple positions every 3 min. GFP fluorescence and DIC images were acquired at each time point with 20- and 2-ms exposure times, respectively (AVI 600 kb)

Video 3 *P.I.* PICH-injected cell. Time-lapse video microscopy showing a representative HeLaS3 cell, stably expressing histone 2B-GFP, injected with anti-PICH antibody (either poly- or monoclonal). Images were acquired at multiple positions every 3 min. GFP fluorescence and DIC images were acquired at each time point with 20- and 2-ms exposure times, respectively (AVI 697 kb)

Video 4 siGl2-transfected cell. Time-lapse video microscopy showing a representative HeLaS3 cell, stably expressing histone 2B-mCherry, transfected with Gl2 (control) siRNA. Images were acquired at multiple positions every 3 min. mCherry fluorescence and DIC images were acquired at each time point with 20- and 2-ms exposure times, respectively (AVI 922 kb)

Video 5 siPICH-transfected cell. Time-lapse video microscopy showing a representative HeLaS3 cell, stably expressing histone 2B-mCherry, transfected with PICH-directed siRNA. Images were acquired at multiple positions every 3 min. mCherry fluorescence and DIC images were acquired at each time point with 20- and 2-ms exposure times, respectively (AVI 807 kb)

Video 6 Rescue of PICH siRNA by PICH-WT. Time-lapse video microscopy showing a representative HeLaS3 cell, stably expressing histone 2B-mCherry, transfected with PICH-directed siRNA and a siRNA refractory plasmid coding for PICH-WT. Images were acquired at multiple positions every 3 min. GFP and mCherry fluorescence images were acquired at each time point with 20-ms exposure times (AVI 773 kb)

Video 7 Lack of rescue of PICH siRNA by PICH-WAB. Time-lapse video microscopy showing a representative HeLaS3 cell, stably expressing histone 2B-mCherry, transfected with PICH-directed siRNA and a siRNA refractory plasmid coding for ATPase-dead PICH (PICH-WAB). Images were acquired at multiple positions every 3 min. GFP and mCherry fluorescence images were acquired at each time point with 20-ms exposure times (AVI 1637 kb)

Video 8 Rescue of PICH siRNA by PICH-TA. Time-lapse video microscopy showing a representative HeLaS3 cell, stably expressing histone 2B-mCherry, transfected with PICH-directed siRNA and a siRNA refractory plasmid coding for a PICH mutant that is unable to bind Plk1 (PICH-TA). Images were acquired at multiple positions every 3 min. GFP and mCherry fluorescence images were acquired at each time point with 20-ms exposure times (AVI 559 kb)

Video 9 Lack of rescue of PICH siRNA by PICH-WAB-TA. Time-lapse video microscopy showing a representative HeLaS3 cell, stably expressing histone 2B-mCherry, transfected with PICH-directed siRNA and a siRNA refractory plasmid coding for a ATPase-dead PICH that is unable to bind Plk1 (PICH-WAB-TA). Images were acquired at multiple positions every 3 min. GFP and mCherry fluorescence images were acquired at each time point with 20-ms exposure times (AVI 1198 kb)
